# Future Trends in Endodontics: How Could Materials Increase the Long-Term Outcome of Root Canal Therapies?

**DOI:** 10.3390/ma15103473

**Published:** 2022-05-12

**Authors:** Alessio Zanza, Rodolfo Reda, Francesco Pagnoni, Shankargouda Patil

**Affiliations:** 1 Department of Oral and Maxillo-Facial Sciences, Sapienza, University of Rome, 00161 Rome, Italy; rodolfo.reda@uniroma1.it (R.R.); francesco.pagnoni22@gmail.com (F.P.); 2 Department of Maxillofacial Surgery and Diagnostic Sciences, Division of Oral Pathology, College of Dentistry, Jazan University, Jazan 45412, Saudi Arabia; drravipatil@gmail.com

The goals of endodontic therapies are the prevention or the elimination of apical periodontitis of endodontic origin, ensuring the stability of results over time in order to avoid the recurrence of the disease and preventing teeth from requiring extraction [1].

Different systematic reviews have been performed with the aim of evaluating the factors influencing the outcome of primary root canal treatments [2,3,4]. Generally, the reasons for the immediate or delayed failure of endodontic therapies are fundamentally related to the following variables: insufficient mechanical instrumentation, inadequate chemical disinfection of canals, and a low quality of root canal filling and post-treatment restoration [4]. All of these problems lead to a non-resolution of the causative agents of endodontic infection, the bacteria. In fact, the most significant reasons for primary root canal treatment (RCT) failure are their persistence and their secondary contamination of the root canal system, which could cause recurrent disease with the exacerbation of symptoms [4]. Nevertheless, the recurrent infection-related aspects are not the unique cause of long-term failure, since the mechanical fracture of endodontically treated teeth can occur [5,6]. As thoroughly demonstrated, the post-endodontic restoration of teeth with crowns or cast restorations does not influence treatment success based on periapical healing as long as there is no sign of coronal leakage; however, it can influence the survival rate of teeth over time, reducing the mechanical failure rate [7,8]. Despite this, the literature findings are still unclear regarding the relationship between the long-term outcome of RCT and the post-endodontic treatment plan with regard to the material of choice [9,10]. Moreover, in recent years, the development of new technologies has improved the mechanical and the metallurgical performance of restorative materials, giving to clinicians a wide range of choice [11]. The selection of the most adequate material is essential for the outcome of post-endodontic restoration for two main reasons. Firstly, the restoration should not interfere with the mechanical load of the tooth, protecting it from fractures arising from occlusal loads [6]. Secondly, the material should guarantee an intrinsic integrity and a good marginal adaptation over time in order to avoid leakage that could cause bacteria infiltration and the secondary contamination of the root canal system [7]. However, the most recent available data on the survival and failure rates of endodontically treated teeth according to the material used for the post-endodontic restoration in relation to the prosthodontic plan are still ambiguous, and clinicians often develop the treatment plan based on their personal judgment rather than on scientific evidence [12].

Regarding the incomplete chemo-mechanical disinfection of the root canal system, the most significant affecting factors are undoubtedly the missed instrumentation of root canals, the impossibility to reach the working length, the alteration of the anatomy or the fracture of nickel–titanium rotary instruments inside the canals [4]. The presence of an instrument fragment is not an intrinsic cause of RCT failure; however, it can increase its percentage because of the increased likelihood of leaving bacteria and/or endodontic tissues inside the root canal system [13]. As demonstrated in the literature, the main causes of the intracanal separation of NiTi rotary instruments are cyclic fatigue, excessive torsional loads, or the combination of these two factors [13]. Recently, the knowledge of the factors influencing the mechanical resistance of endodontic instruments has been expanded, but the dynamic interaction between flexural and torsional stresses remains unclear. In fact, it has demonstrated that the cyclic fatigue resistance is determined by the mass and the crystallographic phase of the instrument [14]. On the contrary, the torsional resistance is not determined by the mass in terms of absolute value, but instead by its distribution in relation to the centre of rotation [15]. As stated by Zanza et al., it can be resumed with the concept of the polar moment of inertia. Moreover, the static interaction between flexural stress and the torsional resistance of instruments has been assessed, showing that increasing the flexural moment acting on tools causes the resistance to increase [16,17,18]. However, the studies published on this topic are only a static evaluation of the interaction of both stresses; thus, an in-depth comprehension of the dynamic relationship between flexural and torsional moments is needed, considering the cutting action of instruments during the shaping procedures.

In conclusion, despite the improvement in the last decade in our knowledge of the performance of endodontic materials, both for instrumentation procedures and post-endodontic restoration, an in-depth comprehension of their mechanical behaviour is still required, and further research is needed both to enhance the success rate of RCT and to improve the performance and quality of materials.
